# Forest Before Trees: Letter Stimulus and Sex Modulate Global Precedence in Visual Perception

**DOI:** 10.3389/fpsyg.2021.546483

**Published:** 2021-03-24

**Authors:** Andrea Álvarez-San Millán, Jaime Iglesias, Anahí Gutkin, Ela I. Olivares

**Affiliations:** Department of Biological and Health Psychology, Faculty of Psychology, Universidad Autónoma de Madrid, Madrid, Spain

**Keywords:** global precedence, hierarchical figures, menstrual cycle, sex differences, visual attention, visual perception, global/local processing, Navon task

## Abstract

The global precedence effect (GPE), originally referring to processing hierarchical visual stimuli composed of letters, is characterised by both global advantage and global interference. We present herein a study of how this effect is modulated by the variables letter and sex. The Navon task, using the letters “H” and “S,” was administered to 78 males and 168 females (69 follicular women, 52 luteal women, and 47 hormonal contraceptive users). No interaction occurred between the letter and sex variables, but significant main effects arose from each of these. Reaction times (RTs) revealed that the letter “H” was identified more rapidly in the congruent condition both in the global and the local task, and the letter “S” in the incongruent condition for the local task. Also, although RTs showed a GPE in both males and females, males displayed shorter reaction times in both global and local tasks. Furthermore, luteal women showed higher *d’* index (discrimination sensitivity) in the congruent condition for the local task than both follicular women and hormonal contraceptive users, as well as longer exploration time of the irrelevant level during the global task than males. We conclude that, according to the linear periodicity law, the GPE is enhanced for compound letters with straight vs. curved strokes, whereas it is stronger in males than in females. Relevantly, luteal phase of the menstrual cycle seems to tilt women to rely on finer grained information, thus exhibiting an analytical processing style in global/local visual processing.

## Introduction

Previous literature has established that in visual perception the global processing of a visual scene precedes the analysis of its local components. While Kinchla (1974) pioneered the use of hierarchical stimuli to evaluate “multielement stimulus arrays” for the presence of certain critical elements, the global precedence effect (GPE) was described by Navon (1977). Navon used a directed attention task with hierarchical stimuli, initially defined by large letters “H” and “S” made up of small letters “_H_” and “_S_.” The global and the local letters were either congruent (e.g., global “S” and local “_S_”) or incongruent (e.g., global “H” and local “_S_”). Participants were asked to identify (a) the global stimulus while ignoring the local elements (global task) and (b) the local elements while ignoring the global shape (local task) in two different blocks. Global precedence was evidenced by faster global than local responses in the congruent trials (global advantage) and slower identification of local targets in the presence of global distractors in the incongruent trials (global interference). Poirel et al. (2008) suggested that the global advantage emerges early in perception whereas the global interference occurs at a later stage.

Experimental studies following Navon (see Kimchi, 2015 for a review) underscored the need to control certain procedural variables that modulate the GPE. Thus, it is important that the presentation of the hierarchical stimulus is randomly assigned to any of the visual quadrants or hemifields on the screen, as certainty regarding the central presentation of the stimulus attenuates global processing (Grice et al., 1983; Lamb and Robertson, 1988). Moreover, when the eccentricity of both levels is not equated, the overall visual angle of the hierarchical stimulus should not exceed 7°-9°. A larger sized stimulus would imply the displacement of global information toward the periphery of the retina while information at the local level would remain close to the fovea, favouring local processing (Kinchla and Wolfe, 1979; Navon and Norman, 1983; Lamb and Robertson, 1990). Regarding the exposure time for each hierarchical stimulus, it was observed that while global advantage remains constant independently of exposure time to the stimulus, interference is affected. That is, in addition to global interference, local interference occurs as stimulus exposure time increases. This phenomenon is called bidirectional (i.e., global-to-local and local-to-global) interference (Paquet and Merikle, 1984; Luna, 1993). The possible modulation of the GPE by exposure time should be taken into account given that this variable differs greatly among studies, ranging from brief presentations (e.g., 40 ms, Navon, 1977) to presentations of unlimited duration (Agnew et al., 2016), with a predominance of exposure times from 100 to 200 ms. It has also been demonstrated that global advantage decreases with increasing sparsity of stimuli (Martin, 1979a), indicating that stimuli should be configured as similarly as possible (Staudinger et al., 2011). Little research has been conducted regarding other variables that influence global/local processing, particularly sex, or specific letter stimuli used, which are focused on in the present study.

In a recent meta-analysis Rezvani et al. (2020) point out that those variables influencing the global/local visual processing can be divided into two major categories: external factors called perceptual field variables (PFVs; e.g., stimulus size, eccentricity, sparsity, and shape type) and subjective or internal/individual factors (e.g., age, disorder, and culture). In the present study we are addressing one type of variable accounting for each of these two major categories. On the one hand, letters “H” and “S,” those used as stimuli in the classical Navon task and shaped by straight strokes and curved lines, respectively, are used in our design as a PFV factor that might modulate the GPE. On the other hand, we examined sex differences as well as hormonal status in females as internal factors influencing the GPE, taking into account that sex-related processing styles in a variety of complex cognitive tasks are associated to basic global/local processing at the attentional level (Pletzer et al., 2017).

Regarding the letter factor, despite notable differences among studies in relation with the composition of hierarchical stimuli using letters, the effect that letters per se have on the Navon task has rarely been examined and results are still inconclusive. Moreover, different letter-forms mean that composing a large letter using small ones causes differences in the physical appearance of the letter which needs to be taken into account. In this respect, some authors (e.g., Gable et al., 2013) have opted for maintaining a constant number of elements in the composition of the global hierarchical stimulus, thus giving rise to varying degrees of sparsity between the vertical and horizontal strokes in global letters (cf. stimuli used by Gable et al., 2013). Following Martin (1979a) and Staudinger et al. (2011), dense strokes facilitate global processing and more disperse strokes facilitate local processing; therefore, heterogeneous density in the strokes composing the stimulus may notably alter its identification. By contrast, most authors have maintained the same configuration for global stimuli, but varying the number of elements composing each letter (cf. stimuli used by Gerlach and Starrfelt, 2018). In any event, the effect of the letter in the Navon task is insufficiently defined. Lamb and Robertson (1988) were the first, using the letters “H” and “S” composed of 12 and 14 elements, respectively, to find an unexpected effect of the identity of the letter used, characterised by smaller RTs to the target letter “H” than to the target letter “S” during the global task. This result was replicated 2 years later (Lamb et al., 1990) with the letters “H” and “S” composed of 13 and 17 elements, respectively. Lamb and Robertson (1988) also found greater global interference during the local task when the target letter was “_H_” (global “S” and local “_H_,” S_H_) than when the target letter was “_S_” (global “H” and local “_S_,” H_S_). These authors suggested that local letters “_S_” may be more salient than local letters “_H_,” and that for this reason the local processing of the stimulus H_S_ generated less global interference. However, other studies by Lamb and Robertson (1989) and subsequently by Giersch et al. (1997) found no differences in RTs for the letters “H” and “S” (in both studies composed of 13 and 17 elements, respectively). Moreover, Giersch et al. (1997) did observe in a second experiment with the target letters “F” and “U” that the local letter “_F_” was identified more rapidly than the local letter “_U_,” while no differences were observed in the identification of global letters.

Even though the above mentioned studies underscored the importance of letter identity in the Navon task, from the scarce experimental evidence available no clear conclusion can be drawn on its influence on the GPE. In consequence, one of the aims of the present work was to analyse whether the letters “H” and “S,” defined with identical size and differing minimally in the number of elements owing to their different letter form, modulate global/local processing differently. In particular, our hypothesis was that large letters “H” (H_H_ and H_S_) can be processed more quickly than large letters “S” (S_S_ and S_H_), bearing in mind the different predominance of straight versus curved strokes in each case. This idea is inspired by the linear periodicity law (Uttal, 1975), according to which the visual system displays greater sensitivity for detecting straight vs. curved lines conforming visual objects.

Additionally, the GPE appears to be modulated to some extent by sex (see Herrera et al., 2019 for a review). In former studies, modulation of the GPE by internal/individual factors was already identified. For example, Lachmann et al. (2014) showed that the GPE disappears for letters if the visual angle mimics conditions of reading of individual letters, while it remains for non-letters. However, later, Schmitt et al. (2019) found that this design effect depends on children’s reading skills. With regard to the subjective variable of interest here (i.e., sex), earlier studies applying the Navon task found smaller RTs in men than in women, both in directed (Martin, 1979b) and in divided (Lee et al., 2012) attention tasks, the latter requiring simultaneously global and local attention. As for possible interactions between sex and level of attention, the literature reveals that global level processing during directed attention tasks is easier for males (Razumnikova and Volf, 2011) and that local level processing is easier for women during both directed (Müller-Oehring et al., 2007) and divided (Roalf et al., 2006) attention tasks.

To better understand the possible biological factors underlying the GPE, differences between men and women were analysed recently, taking into account the different phases in women’s menstrual cycle, with inconclusive results in the same research group applying the Navon task. More specifically, Pletzer et al. (2014) reported that women in the luteal phase showed a smaller global advantage than males, women in the follicular phase and contraceptive users. It is known that the level of progesterone is higher during the luteal phase than in the follicular phase of the menstrual cycle (Reed and Carr, 2018). Pletzer et al. (2014) found a negative correlation between the levels of progesterone and global advantage, thus explaining the attenuation in global advantage encountered in luteal women. This correlation was likewise encountered by Pletzer and Harris (2018) in a sample of males and luteal females, whereas Scheuringer and Pletzer (2016) did not find any correlation related to progesterone levels. In subsequent studies, however, Pletzer et al. (2017) and Pletzer and Harris (2018) did not find differences between sexes or between menstrual cycle phases in the Navon task. In order to determine the importance of sex in this field of research, another of the aims of the present work was to analyse the differences between males and females during global/local processing. This analysis was carried out taking into account women’s hormonal status depending on their menstrual cycle phase or whether they are contraceptive treatment users. In concrete terms, we hypothesised that males would show a stronger global precedence effect than females, and that luteal women would reveal an attenuated global advantage when compared to other female groups, supporting that sex differences in executing the Navon task can be associated to different hormonal statuses of women in relation with reproductive functions.

## Materials and Methods

### Participants

In response to a public call launched on the Faculty of Psychology webpage, 265 University students of ages from 17 to 29 years volunteered to participate. After signing their declaration of informed consent, the female participants completed a questionnaire on their menstrual cycle in which they indicated the average duration of their cycle (time elapsed between periods), the number of days elapsed since the beginning of their last period, and whether they were taking hormonal contraceptives. This information established the phase at which each participant was in her menstrual cycle, as per Fehring et al. (2006). After excluding 19 women whose menstrual cycle was irregular (i.e., less than 21 or more than 35 days), mean cycle length of the remaining 168 women was 28.9 days (*SD*: 2.31). The final sample comprised 246 participants (*M* = 19.5; *SD* = 1.73): 78 were male and 168 female, of which 69 women were in their follicular phase, 52 women in their luteal phase, and 47 were hormonal contraceptive users.

Participants completed the Edinburgh Handedness Inventory (Oldfield, 1971) and the State-Trait Anxiety Inventory (STAI: Spielberger et al., 1983). We included the STAI scales bearing in mind the study by Derryberry and Reed (1998) on the relationship between anxiety and narrowing of the attentional focus during visual processing of hierarchical stimuli. Subsequently, they performed a range of behavioral tasks (these were presented in a counterbalanced order). The present study addresses the results relative to the Navon task using the letters “H” and “S.” Table 1 shows a summary of the characteristics of each of the four groups of participants considered in this study, taking into account the number of participants, their age and mean score on the handedness, state and trait anxiety scales. All participants were rewarded with academic credits for their participation, and the Ethics committee of the University approved the study (Code: CEI-71-1271).

**TABLE 1:** Characteristics of the groups studied.

	**Male**	**Follicular women**	**Luteal women**	**Contraceptive users**
*N Total*	78	69	52	47
*Age* (SD)	19.3 (1.35)	19.4 (1.81)	19.8 (2.09)	19.8 (1.68)
*Handedness* (SD)	1.89 (0.82)	1.89 (0.85)	1.92 (0.87)	1.85 (0.85)
*State STAI* (SD)	15.7 (9.23)	16.6 (8.67)	16.4 (7.96)	15.9 (8.04)
*Trait STAI* (SD)	21.8 (11.1)	22.0 (10.8)	22.9 (8.69)	24.6 (8.82)

### Apparatus and Stimuli

Participants were seated at separate computer workstations within a soundproofed and well-illuminated room, at a distance to the screen of approximately 70 cm. The stimuli were presented in black (3.19 cd/m^2^) on a white background (192.8 cd/m^2^), on 16-inch screens using the software “Estimulador Cognitivo” (Neuronic S.A.). As applied by other authors (Behrmann et al., 2005; Duchaine et al., 2007a,b; Busigny and Rossion, 2011), the stimuli used were large letters “H” and “S” composed of small letters “_H_” or “_S_.” The large letters measured 9 cm in height and 6.5 cm in width (7.36°× 5.32° vertical and horizontal visual angles, respectively), while the small letters measured 1.30 cm in height and 1 cm in width (1.06°× 0.82° vertical and horizontal visual angles, respectively). The hierarchical letters “H” and “S” were of identical size, but composed of a different number of elements, as the characteristic curved strokes of the letter “S” required a larger number of elements than the straight strokes of the letter “H.” The letter “H,” therefore, was composed of 12 elements, while the letter “S” was composed of 14 elements.

**FIGURE 1:**
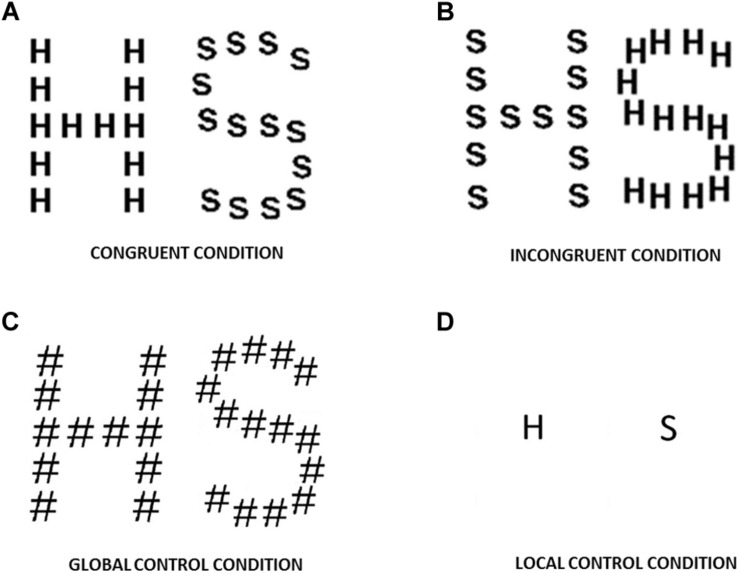
Stimuli used: **(A)** congruent condition; **(B)** incongruent condition; **(C)** control stimuli for the global task; **(D)** control stimuli for the local task. Note the different letter-form (straight vs. curved strokes in the letters “H” and “S,” respectively) and the different number of elements in each large letter (12 elements in the letter “H” and 14 in the letter “S”).

The three experimental conditions set for the presentation of these letters were: congruent, incongruent and control. In the congruent condition, the same letter was presented as a large letter and small letters. In the incongruent condition, the large letter and small letters were different. In the control condition, only a large or a small letter was visible, depending on whether a global or a local directed attention task was performed. The control stimuli in the global task were the large letters “H” and “S” made up of the symbol “#” (which has no alphabetic meaning for native European Spanish people). According to other authors (Lamb and Robertson, 1988; Roux and Ceccaldi, 2001; Agnew et al., 2016), the control stimuli for the local task were the letters “_H_” and “_S_” presented singly in the same size as the elements used for the hierarchical shapes, in order to determine whether small letters were processed differently when they form part or not of a compound stimulus. The stimuli and different experimental conditions are illustrated in Figure 1.

### Procedure

The stimuli were administered in two blocks of 120 trials each, on one occasion asking participants to attend selectively to the large letter (global task/level) and on another to attend selectively to the small letter (local task/level). Participants were asked to respond as quickly and accurately as possible stating whether the letter displayed on the screen for the level attended was “H” or “S.” They registered their answers pressing with their dominant hand the left mouse button when the letter attended to was an “H” and the right mouse button if the letter was an “S.”

Half of the participants completed the directed attention tasks in the order global-local, and the other half in the order local-global. Each global or local task was preceded by the corresponding instructions and 12 practice trials. Until the participant had understood and was proficient at the practice trials, the experiment did not commence. Subsequently, a reminder was given of the instructions indicating that the global or local task, as appropriate, was about to begin. Stimuli appeared randomly during 200 ms on any sector of the screen (top-left, top-right, bottom-left, and bottom-right), with an ISI of 1000 ms during which the participant had to answer. The number of congruent, incongruent and control trials was equivalent, and their order of appearance random for each block. Similarly, the appearance of the letters “H” or “S” and the sector of the screen in which the stimuli were presented were random.

### Data Analysis

Having verified variance homogeneity by means of Levene’s test, an ANOVA was conducted in which the null hypothesis was maintained for the absence of differences (F < 1) among the groups on the handedness, state anxiety, and trait anxiety scales. Consequently, it was not necessary to use the results on such scales as co-variables in the analyses described below.

The RTs for correct answers were measured with a mixed general linear model with three fixed within-participants factors and one fixed between-participants factor, establishing a diagonal variance-covariance matrix. The independent variables were the within-participants factors *Task* (global/local), stimulus *Congruency* (congruent/incongruent/control) and target *Letter* (“H”/“S”), in addition to the between-participants factor *Sex* (males/females). To compare the findings of this study against previous literature on hormonal differences in the Navon task, this same analysis was also conducted substituting the between-participants factor *Sex* with the factor *Hormonal status* (males/follicular women/luteal women/contraceptive users).

Two adjustments were made to the model. The more complex of these comprised four-fold interaction (*Task* × *Congruency* × *Letter* × *Sex/Hormonal Status*), while in the more restricted adjustment this interaction was eliminated. The likelihood ratio for these two models did not evidence significant differences of fit (*p* = 0.99); consequently, applying the parsimony criterion, we selected the least restricted model, i.e., with the fewest parameters. Five values were found to have an elevated positive skewness of the residual (i.e., abnormally high RTs), most likely due to distractions at the moment of issuing a response and, therefore, residuals were categorised and extreme atypical cases were eliminated (−3 < Z_Residual_ < 3), following the procedure described by Pardo and San Martín (2015, cf. Supplementary Material_Data analysis).

To analyse the between-participants factor *Sex/Hormonal status*, the discriminability index *d’* was also calculated. This, according to the signal detection theory (Green and Swets, 1966), reveals the ease with which participants could differentiate the letters “H” and “S” in each task and experimental condition. The *d’* index was calculated using the formula described in Meyer et al. (1988), where pH denotes the mean probability of generating hits and pFA denotes the mean probability of generating false alarms:

d=′  0.6logep⁢H⁢(1-p⁢F⁢A)p⁢F⁢A⁢(1-p⁢H)

Hit and false alarm probabilities of 1 or 0 were adjusted to 0.999 or 0.001, respectively, according to MacMillan and Creelman (2005). The *d’* index for each condition was calculated according to the response categories shown in Table 2.

**TABLE 2:** Response categories for calculating *d’.*

**GLOBAL**			**LOCAL**		
Congruent	Hits	Correct H_H_	Congruent	Hits	Correct H_H_
	Fails	Incorrect H_H_		Fails	Incorrect H_H_
	False alarms	Incorrect S_S_		False alarms	Incorrect S_S_
	Correct rejections	Correct S_S_		Correct rejections	Correct S_S_
Incongruent	Hits	Correct H_S_	Incongruent	Hits	Correct S_H_
	Fails	Incorrect H_S_		Fails	Incorrect S_H_
	False alarms	Incorrect S_H_		False alarms	Incorrect H_S_
	Correct rejections	Correct S_H_		Correct rejections	Correct H_S_

The *d’* index was analysed by means of a mixed general linear model with two fixed within-participants factors and one fixed between-participants factor, establishing a diagonal variance-covariance matrix. The independent variables were the within-participants factors *Task* (global/local) and stimulus *Congruency* (congruent/incongruent), in addition to the between-participants factor *Sex* (males/females). This same analysis was likewise conducted substituting the between-participants factor *Sex* for the *Hormonal status* (males/follicular women/luteal women/contraceptive users) factor.

In both measures (RTs and *d’*) a comparison was drawn using the Tukey’s *post hoc* pairwise comparison test, applying the Bonferroni correction for significant effects. The results are reported using Fisher’s *F-test*. Statistical significance was established with a confidence level of 95%. In accordance with Cohen (1988), the effect size was estimated by calculating eta-squares (η^2^). Speed-accuracy trade-off was measured by correlating the RTs and the *d’*.

## Results

The RT analysis showed significant effects for *Task F*(1,2850) = 460, *p <* 0.001, η^2^ = 0.14, *Congruency F*(2,1964) = 158, *p <* 0.001, η^2^ = 0.14, *Letter F*(1,2848) = 17.3, *p <* 0.001, η^2^ = 0.006 and *Sex F*(1,2858) = 61.0, *p <* 0.001, η^2^ = 0.021. Similarly, the main effect of the between-participants factor *Hormonal status* proved significant: *F*(3,2846) = 20.9, *p <* 0.001, η^2^ = 0.022. In particular: (a) the global task was performed with shorter RTs than the local task (507 ms < 560 ms); (b) the congruent (*p* < 0.001) and control conditions (*p* < 0.001) were resolved with shorter RTs than the incongruent condition, with no differences found between the first two (*p* = 0.99) (517 ms control, 518 ms congruent, 565 ms incongruent); (c) the target letter “H” was detected more quickly than the target letter “S” (529 ms < 539 ms), and (d) males showed shorter RTs than the rest of women groups (*p* < 0.001), with no differences observed among the women’s groups (*p* = 0.99) (519 ms males, 536 ms luteal women, 538 ms hormonal contraceptive users, 541 ms follicular women). Importantly, we did not find a significant target *Letter*× *Sex/Hormonal status* interaction.

The interaction *Task* × *Congruency* was found to be significant. In particular, for the global task *F*(2,1002) = 39.9, *p <* 0.001, η^2^ = 0.074 the congruent condition (488 ms) was resolved with shorter RTs (*p* < 0.001) than the control (508 ms) and incongruent (526 ms) conditions, and, in turn, the control condition presented shorter RTs (*p* < 0.001) than the incongruent condition. For the local task *F*(2,975) = 172, *p <* 0.001, η^2^ = 0.26, the control condition (527 ms) presented shorter RTs (*p* < 0.001) than the congruent (547 ms) and incongruent (605 ms) conditions, and the congruent condition shorter RTs (*p* < 0.001) than the incongruent condition. The difference between the RTs obtained for the global and local task *F*(2,1304) = 24.1, *p* < 0.001, η^2^ = 0.036 were greater in the incongruent condition than in the congruent (*p* = 0.003) and control (*p* < 0.001) conditions. This fact indicated that the interference effect was greater in the local task (incongruent *minus* congruent = 58 ms) than in the global one (incongruent *minus* congruent = 38 ms). Also, the global/local difference was greater in the congruent condition than in the control condition (*p* < 0.001). The analysis of the *Task* × *Congruency* interaction, by means of the *d’* index, showed that, both in the global task *F*(1,471) = 26.6, *p <* 0.001, η^2^ = 0.054 and the local task *F*(1,447) = 60.0, *p <* 0.001, η^2^ = 0.12, the *d’* index obtained in the incongruent condition (3.90 global and 3.77 local) was smaller than for the congruent condition (5.33 global and 6.00 local).

The interactions *Task* × *Congruency* × *Letter* and *Task* × *Congruency* × *Sex*/*Hormonal status*, relative to the influence exerted by letter and by sex/hormonal group, respectively, were also significant. Table 3 shows the mean values for RT and *d’* per *Task*, *Congruency*, *Letter*, and *Hormonal status*, as well as the results derived from the ANOVAs conducted for each of these measures. Regarding the speed-accuracy trade-off, we found some positive correlations between RTs and *d’* in the *Task* × *Congruency* × *Hormonal status* interaction, as described below.

**TABLE 3:** Results of the ANOVAs comparing RTs and *d’* scores between global/local Navon tasks.

		**Congruent**					**Incongruent**					**Control**				
	**DV**	***M*_G_**	***M*_L_**	***F* (*df1, df2*)**	***p*^c^**	**η^2^**	***M*_G_**	***M*_L_**	***F* (*df1, df2*)**	***p*^*c*^**	**η^2^**	***M*_G_**	***M*_L_**	***F* (*df1, df2*)**	***p*^c^**	**η^2^**
“S”	RT	5037.79	5597.87	98.7 (1, 493)	< 0.001*	0.17	5258.77	5878.53	96.3 (1, 491)	< 0.001*	0.16	5228.00	5358.02	4.81 (1, 492)	0.029*	0.010
“H”	RT	4747.47	5368.58	114 (1, 484)	< 0.001*	0.19	5268.42	6239.03	234 (1, 489)	< 0.001*	0.32	4938.71	5197.30	2.8 (1, 478)	< 0.001*	0.042
Males	RT	4789.54	53010.2	53.8 (1, 961)	< 0.001*	0.053	50910.8	58710.9	99.5 (1, 963)	< 0.001*	0.094	49110.5	5179.48	12.3 (1, 954)	< 0.001*	0.013
	*d*’	5.710.70	6.660.78	3.10 (1, 471)	0.079	–	4.160.64	3.690.60	1.11 (1, 474)	0.29	–					
Follicular women	RT	49110.0	56010.9	84.4 (1, 958)	< 0.001*	0.081	52911.2	61511.7	108 (1, 962)	< 0.001*	0.10	51410.9	53710.2	9.57 (1, 957)	0.002*	0.010
	*d*’	5.400.75	5.310.82	.026 (1, 471)	0.87	–	4.040.67	3.810.63	.26 (1, 474)	0.61	–					
Luteal women	RT	49311.6	54812.5	39.8 (1, 959)	< 0.001*	0.040	53613.2	60313.3	49.5 (1, 962)	< 0.001*	0.049	51312.7	52611.6	2.38 (1, 953)	0.12	–
	*d*’	5.930.86	7.130.95	3.33 (1, 471)	0.069	–	3.850.78	3.740.73	0.041 (1, 474)	0.84	–					
Contrac. users	TR	49212.1	55113.1	42.3 (1, 958)	< 0.001*	0.042	52913.6	61314.0	71.5 (1, 962)	< 0.001*	0.069	51313.3	52912.2	3.00 (1, 954)	0.084	–
	*d*’	4.250.90	4.890.99	0.86 (1, 471)	0.36	–	3.530.81	3.860.76	0.34 (1, 474)	0.56	–					

± 1.96⋅SE of the mean is shown below of each value. Target *Letter*: “S”, “H”; *Hormonal Status*: males, follicular women, luteal women, hormonal contraceptive users (Contrac. users); Stimulus *Congruency*: congruent, incongruent, control; *M*_G_, Estimated mean for the global task; *M*_L_, Estimated mean for the local task. ^*c*^Bonferroni correction for several comparisons. * Statistically significant differences (*p* < 0.05).

### Letter Stimulus

The interaction *Task* × *Congruency* × *Letter* revealed shorter RTs in the global task in comparison with the local task for both target letters and in all conditions. When we analysed each letter separately, for the letter “H” *F*(2,907) = 34.1, *p* < 0.001, η^2^ = 0.070, the difference between RTs obtained for the global and local tasks was greater in the incongruent condition than in congruent (*p* < 0.001) and control (*p* < 0.001) conditions, as well as in the congruent condition than in control one (*p* < 0.001). As for the letter “S” *F*(2,443) = 6.11, *p* = 0.002, η^2^ = 0.027, this difference between global and local tasks was greater in the congruent (*p* = 0.002) and incongruent (*p* = 0.001) conditions than in the control one, but the difference was similar between congruent and incongruent conditions (*p* = 0.45). That is, for the letter “H” the interference effect was greater in the local task (incongruent *minus* congruent = 87 ms) than in the global one (incongruent *minus* congruent = 52 ms), but the global/local interference was similar for the letter “S” (incongruent *minus* congruent = 22 ms for global, and = 28 ms for local).

**FIGURE 2:**
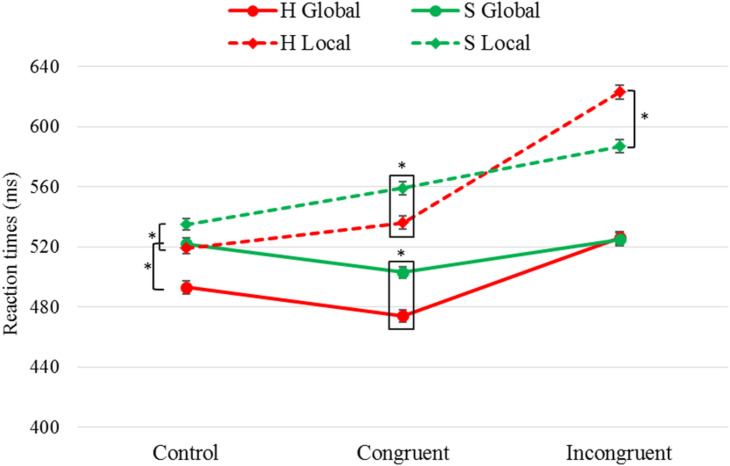
Results of pairwise comparisons of RTs for the factors *Task* × *Congruency* × *Letter*. *Task*: global, local; target *Letter*: “H”, “S”; stimulus *Congruency*: congruent, incongruent, control; ^∗^statistically significant differences (*p* < 0.05). Error bars show standard error of the mean. Note that the letter “H” was associated with shorter RTs in both tasks for the control and congruent conditions. In the incongruent condition, no differences were found between these two letters in the global task, while in the local task the letter “S” obtained shorter RTs.

Table 4 shows pairwise comparisons between the target *Letter* taking into account *Task* and *Congruency*, represented graphically in Figure 2. We found shorter RTs when the target letter was “H” (vs. “S”) in both tasks and for both congruent and control conditions. However, for the incongruent condition we observed shorter RTs when the target letter was “S” (vs. “H”) only for the local task; that is, greater interference was found when the local target letter was “_H_” (S_H_) than when it was “_S_” (H_S_). Finally, it was seen that, both in the global task *F*(2,1000) = 8.21, *p <* 0.001, η^2^ = 0.016 and in the local task *F*(2,973) = 27.1, *p <* 0.001, η^2^ = 0.053, the difference between the RTs obtained for the target letters “H” and “S” were greater in the congruent and control conditions than in the incongruent condition, while no differences we found between the first two.

**TABLE 4:** Results of pairwise comparisons of *Task* × stimulus *Congruency* × target *Letter* comparing mean RTs between *Letters* “H” and “S”.

		***F* (*df1, df2*)**	***p***	**η^2^**
Global	Congruent	27.5 (1, 479)	<0.001^†^	0.054
	Incongruent	0.02 (1, 477)	0.089	–
	Control	24.4 (1, 477)	<0.001^†^	0.049
Local	Congruent	15.1 (1, 481)	<0.001^†^	0.031
	Incongruent	33.0 (1, 483)	<0.001^‡^	0.64
	Control	8.23 (1, 481)	0.004^†^	0.017

*^†^*p* ≤ 0.05 RTs for “H” < for “S”. ^‡^*p* ≤ 0.05 RTs for “S” < for “H.”*

### Sex and Hormonal Status

Regarding the interaction *Task* × *Congruency* × *Sex*, shorter RTs were found in the global task as opposed to the local task in men as in women in every condition [*Δ*_G–L_ Congruent: females *F*(1,963) = 165, *p <* 0.001, η^2^ = 0.15, males *F*(1,965) = 53.9, *p <* 0.001, η^2^ = 0.053; *Δ*_G–L_ Incongruent: females *F*(1,966) = 226, *p <* 0.001, η^2^ = 0.19, males *F*(1,967) = 100, *p <* 0.001, η^2^ = 0.093; *Δ*_G–L_ Control: females *F*(1,961) = 14.0, *p <* 0.001, η^2^ = 0.014, males *F*(1,958) = 12.3, *p <* 0.001, η^2^ = 0.013]. Additionally, both females *F*(2,1241) = 43.9, *p* < 0.001, η^2^ = 0.066 and males *F*(2,392.4) = 6.43, *p* = 0.002, η^2^ = 0.032 showed differences between the RTs obtained for the global and local tasks, revealing greater differences in the incongruent condition than in the congruent (females *p* = 0.027; males *p* = 0.017) and control (females *p* < 0.001; males *p* = 0.001) conditions. Also, differences between RTs were greater in the congruent condition than in the control one in females *p* < 0.001, but not in males *p* = 0.18. That is, for both females and males the interference effect was greater in the local task (incongruent *minus* congruent = 55 ms for females, and = 57 ms for males) than in the global one (incongruent *minus* congruent = 25 ms for females, and = 31 ms for males).

**FIGURE 3:**
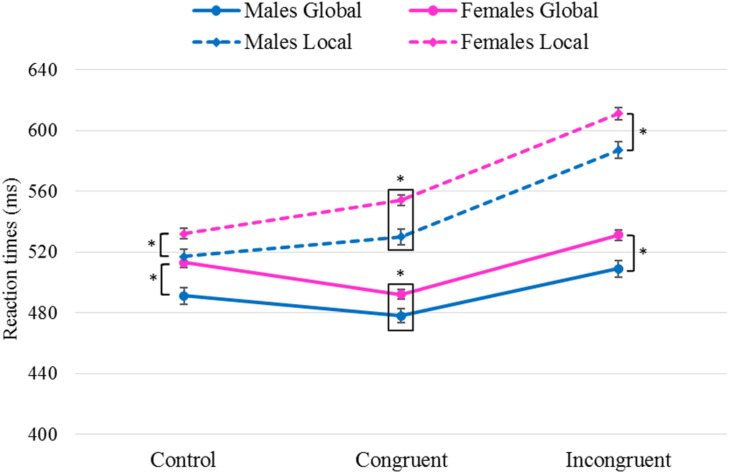
Results of pairwise comparisons of RTs for the factors *Task*× *Congruency*× *Sex*. *Task*: global, local; *Sex*: males, females; stimulus *Congruency*: congruent, incongruent, control. *Statistically significant differences (*p* < 0.05). Error bars show standard error of the mean. Note that men exhibited shorter RTs than women in all tasks and conditions.

Figure 3 shows the significant differences found as a function of the between-participants *Sex* variable. In all conditions and in both tasks, males presented shorter RTs than females [*Δ*_Males–Females_ Congruent: global *F*(1,481) = 5.63, *p* = 0.018, η^2^ = 0.012, local *F*(1,483) = 14.3, *p <* 0.001, η^2^ = 0.029; *Δ*_Males–Females_ Incongruent: global *F*(1,479) = 11.0, *p* = 0.001, η^2^ = 0.022, local *F*(1,485) = 12.5, *p <* 0.001, η^2^ = 0.025; *Δ*_Males–Females_ Control: global *F*(1,478) = 11.7, *p* = 0.001, η^2^ = 0.024, local *F*(1,483) = 6.56, *p* = 0.011, η^2^ = 0.013].

When the between-participants variable was *Hormonal status*, all groups showed shorter RTs in the global task than in the local task, both in the congruent and in the incongruent conditions. However, in the control condition, while men and follicular women also had faster RTs in the global task, luteal women and hormonal contraceptive users showed no differences between tasks. Regarding the difference between the RTs obtained for the global and local tasks in each group of females, the follicular *F*(2,514) = 21.1, *p* < 0.001, η^2^ = 0.076, luteal *F*(2,356) = 12.4, *p* < 0.001, η^2^ = 0.065 and contraceptive users *F*(2,345) = 11.7, *p* < 0.001, η^2^ = 0.063 showed a similar difference between congruent and incongruent conditions (*p* > 0.05), revealing a similar interference effect between global (incongruent *minus* congruent = 38 ms for follicular, = 43 ms for luteal, and = 37 ms for contraceptive users) and local (incongruent *minus* congruent = 55 ms for follicular, = 55 for luteal, and = 62 ms for contraceptive users) tasks. Furthermore, the three female groups showed smaller differences in the control condition than in the congruent (follicular *p <* 0.001; luteal *p <* 0.001; contraceptive users *p* = 0.001), and incongruent (follicular *p <* 0.001; luteal *p <* 0.001; contraceptive users *p <* 0.001) conditions.

**FIGURE 4:**
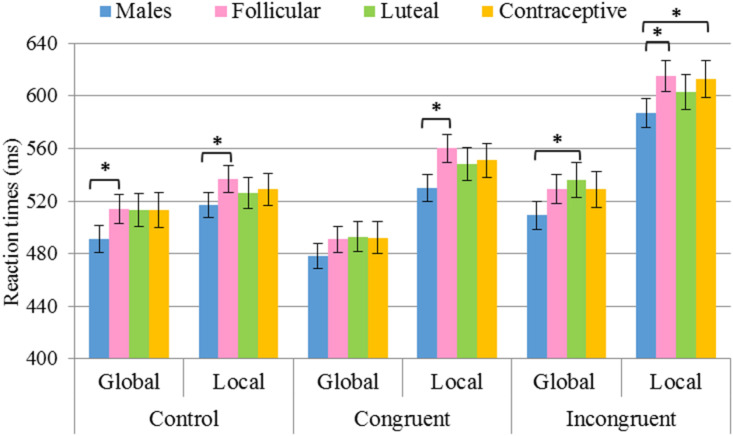
Results of pairwise comparisons of *RTs* for the factors *Task* × *Congruency* × *Hormonal status*. Stimulus *Congruency*: congruent, incongruent, control; *Hormonal status*: males, follicular women, luteal women, hormonal contraceptive users; *Task*: global, local. *Statistically significant differences (*p* < 0.05). Error bars show standard error of the mean.

In comparing the *Hormonal status*, the differences displayed in Table 5 and represented graphically in Figure 4, were observed. In particular, the male group was found to display shorter RTs in the congruent condition for the local task in comparison with the group of follicular women (*p* = 0.001), and shorter RTs in the incongruent condition for the local task vs. the groups of follicular women (*p* = 0.004) and hormonal contraceptive users (*p* = 0.022). Furthermore, shorter RTs in the incongruent condition for the global task were found in the group of males than in the group of luteal women (*p* = 0.014). Differences were likewise found in the control condition, where for both tasks the group of males showed shorter RTs than follicular women (global *p* = 0.022 and local *p* = 0.020).

**TABLE 5:** Results of pairwise comparisons *Task* × stimulus *Congruency*× *Hormonal status* comparing RTs and *d’* scores between *Hormonal status*.

			***F* (*df1, df2*)**	***p***	**η^2^**
Global	Congruent	*RT*	1.91(3,479)	0.127^#^	–
		*d’*	2.83(3,238)	0.039^#^	–
	Incongruent	*RT*	3.90(3,477)	0.009^‡^	0.024
		*d’*	0.54(3,238)	0.66^#^	–
	Control	*RT*	3.90(3,476)	0.009^†^	0.024
Local	Congruent	*RT*	5.51(3,481)	0.001^†^	0.033
		*d’*	5.21(3,238)	0.002^§¶——^	0.062
	Incongruent	*RT*	4.79(3,483)	0.003^†§^	0.029
		*d’*	0.045(3,238)	0.99^#^	–
	Control	*RT*	2.95(3,481)	0.032^†^	0.018

*^†^*p* ≤ 0.05 between males and follicular women. ^‡^*p* ≤ 0.05 between males and luteal women. **^§^***p* ≤ 0.05 between males and hormonal contraceptive users. **^¶^***p* ≤ 0.05 between luteal women and follicular women. **^——^***p* > 0.05 between luteal women and hormonal contraceptive users. **^#^***p* > 0.05 no statistically significant differences on applying Bonferroni correction to pairwise comparisons of levels in the *Hormonal status* factor.*

**FIGURE 5:**
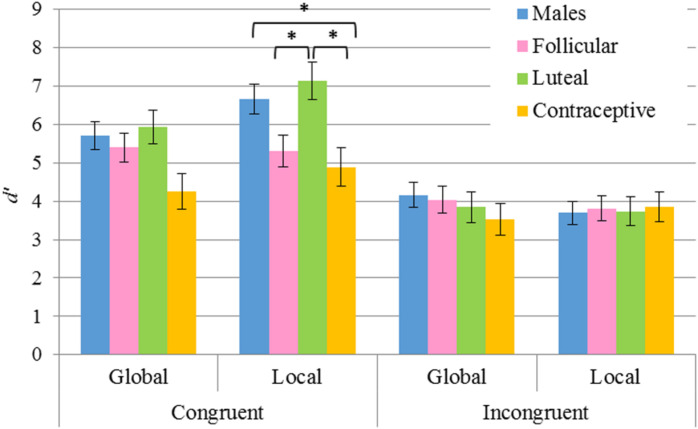
Results of pairwise comparisons of *d’* index for the factors *Task* × *Congruency* × *Hormonal status*. Stimulus *Congruency*: congruent, incongruent; *Hormonal status*: males, follicular women, luteal women, hormonal contraceptive users; *Task*: global, local. *Statistically significant differences (*p* < 0.05). Error bars show standard error of the mean. Note that significant differences according to the *Hormonal status* were only observed in the congruent condition for the local task.

As for the analyses of the *d’* index, the principal factors *Sex F*(1,915) = 3.52, *p* = 0.061 and *Task F*(1,915) = 1.88, *p* = 0.17 were not significant. In addition, the analysis of the interaction *Task* × *Congruency* × *Sex* revealed no differences on comparing by *Task* or by *Sex*. Conversely, the principal factors *Congruency F*(1,915) = 83.8, *p* < 0.001, η^2^ = 0.084 and *Hormonal status F*(3,915) = 4.95, *p* = 0.002, η^2^ = 0.016 were found to be significant. In particular: (a) the congruent condition was discriminated better than the incongruent condition (5.66 > 3.83); and (b) hormonal contraceptive users obtained a smaller *d’* than the males group (*p* = 0.007) and the group of luteal women (*p* = 0.005) (4.13 hormonal contraceptive users, 4.64 follicular women, 5.16 luteal women, 5.05 males). The interaction *Task* × *Congruency* × *Hormonal status* was likewise found to be significant although no differences were found between the global task and the local task. Nevertheless, pairwise comparisons revealed significant differences in performance among the groups in the congruent condition (see Table 5 and Figure 5). Thus, whereas in the global task differences were not significant, in the local task higher *d’* (greater local advantage) was shown by: a) males in comparison to hormonal contraceptive users (*p* = 0.038), and b) luteal women in comparison to follicular women (*p* = 0.031) and to hormonal contraceptive users (*p* = 0.009).

In relation to speed–accuracy trade-off analyses, we found the following positive correlations between RTs and *d’* (i.e., slower responses with better discrimination) for each group: (a) males in the congruent condition for the global task (*r* = 0.35, *p* = 0.002); (b) follicular women in the incongruent condition for the local task (*r* = 0.35, *p* = 0.004); and (c) luteal women in all tasks and conditions (global: congruent *r* = 0.39, *p* = 0.004, incongruent *r* = 0.43, *p* = 0.001; local: congruent *r* = 0.40, *p* = 0.004, incongruent *r* = 0.30, *p* = 0.032). The rest of correlations were not statistically significant.

## Discussion

### The GPE Was Modulated by Letter Stimulus and by Sex/Hormonal Status, but No Interaction Occurred Between These Two Variables

The aim of this study was to determine whether letter (“H” vs. “S”) and sex modulate visual processing of hierarchical stimuli, in a group of males and three groups of females according to their hormonal status. We analysed the RTs obtained in the correct trials as well as the *d’* index for 246 young participants in the Navon task. The RTs clearly showed a global advantage for both target *Letters* used, in all *Sex*/*Hormonal status*. Regarding the interference effect we found a bidirectional interference (i.e., conflicting stimuli in the irrelevant level interfered in both global and local tasks). This result differs from that obtained by Navon (1977), who found a global advantage but a unidirectional interference effect (i.e., limited to a global interference) at 40 ms of exposure duration of stimuli. However, later, several studies have pointed up that, in contrast to the global advantage, the interference effect is very sensitive to the exposure time, finding a bidirectional and asymmetric interference effect from 40 ms of exposure (Luna, 1993). We used an exposure time of 200 ms, which might explain the lack of unidirectional global interference effect in the present study. Thereafter, we will refer to global interference taking into account that this effect was bidirectional (global-to-local and local-to-global) but asymmetric (i.e., greater for global-to-local than for local-to-global) in our case.

No interaction was found between letter and sex. That is, letter processing was neither related to sex nor to any particular menstrual cycle phase or to hormonal contraceptive treatment. Thus, we can consider that each of these variables modulates the GPE independently. These data allow us to clear up some gaps and inconsistencies encountered in previous studies and to offer clarifying information regarding how the Navon task is influenced by letter identity (bearing in mind the inconclusive results in the findings of Lamb and Robertson, 1988, 1989) and by sex depending on the hormonal status of participants (taking into account the inconsistent results reported in Pletzer et al., 2014, 2017). Our findings cannot be explained by participants’ state or trait anxiety, as no differences were found between groups on the State-Trait Anxiety Inventory (STAI). Controlling this variable in studies on the GPE using the Navon task is relevant, as it has been demonstrated that the motivational condition may alter hierarchical visual stimuli processing (Derryberry and Reed, 1998).

Taking into account the results obtained in each different stimulus presentation condition, shorter RTs were obtained in all groups and for each target letter in the congruent condition as compared to the incongruent condition, an effect that is widely accepted in the literature (e.g., Behrmann et al., 2005; Müller-Oehring et al., 2007). However, while the participants performed more rapidly under the congruent condition in the global task, they were faster in the control condition in the local task. These results may be explained by the design of the stimuli we used in the control condition. For the local task, we presented single letters of a small size, as in the study of Roux and Ceccaldi (2001) and Agnew et al. (2016), in order to determine whether small letters were processed differently when they form part or not of a hierarchical stimulus. In effect, our results showed that single letters were identified more rapidly than local letters within a hierarchical compound, which contradicts the results reported by Lamb and Robertson (1988), who found no differences between the congruent and control conditions. Interestingly, a recent multi-voxel pattern analysis-fMRI study (Valdés-Sosa et al., 2020) showed that, whereas level-invariant shape information was found in object and face-selective brain areas, shape-invariant hierarchical level information was found in scene-selective areas. Since hierarchical stimuli integrate both types of information, they would require the activation of independent parallel neural pathways whereas single letters would require the exclusive activation of “shape” areas. This might explain the facilitation of the attention (i.e., shorter RTs) for the control condition in our local task.

As for the global task the control letters were composed of the symbol “#” (which has no alphabetic meaning for native European Spanish people), instead of the letter “O” or the number “0,” as was the case in other studies (Andres and Fernandes, 2006; Agnew et al., 2016). Our aim here was to reduce as far as possible any possible verbal interference on an irrelevant level, with the expectation that these stimuli would elicit shorter RTs than in the congruent condition. The ensuing results showed, however, that the control condition in the global task registered greater RTs than the congruent condition, which is similar to the results obtained by Andres and Fernandes (2006) using the letter “O.” This suggests that the use of a distracting letter or a non-linguistic symbol as elements in the control condition does not alter task performance. We therefore understand that stimuli using the same letter (i.e., congruent) on both levels are processed even more easily than those with minimal interference on the irrelevant level.

Another methodological element to mention regarding the present study is that we did not counterbalance the keys used for participants to give their dichotomous response. Giersch et al. (1997) demonstrated that there was no difference in task performance when participants responded pressing the right or the left key to respond to the letters “H” and “S”, respectively, vs. when the response keys were inverted. These authors replicated this finding in a second experiment using the letters “F” and “U.” Furthermore, our results on the effect of letter coincide with those of Lamb and Robertson (1988), who did counterbalance response hand and keys. On this issue, we discard that differences between letters could be due to a left-right effect such as a SNARC (Spatial Numerical Association of Response Codes) effect of letters. The SNARC effect was originally observed by Dehaene et al. (1993) with numbers in a parity judgment task, in which relatively small numbers were responded to faster with the left hand, whereas relatively large numbers were responded to faster with the right hand. This effect was interpreted as an association between the left and the right side of the egocentric space and the relative position of the number in an analog numerical line. The absence of a preferential response effect in a letter classification task led to the authors to conclude that the spatial congruence effect is specific to the numerical domain. Although Gevers et al. (2003) reported later a spatial congruency effect with letters of the alphabet, more recently Dodd et al. (2008) found that letters induced spatial shifts of attention only when the processing of ordinal information was task relevant (see Santiago et al., 2011, for a review). In spite of such findings, we do consider the lack of counterbalancing the response keys as a limitation in the present study that narrows the scope of our conclusions. Thus, we suggest bearing in mind this procedural aspect in future experiments in order to discard possible stimulus-response side effects.

In what follows, we discuss separately the findings for the letter and sex/hormonal status variables, both of which modulate the GPE independently.

#### Modulating Effect of Letter Stimulus on Navon Task Performance

In earlier studies using letters as hierarchical visual stimuli it has been widely shown that the GPE is produced independently of the letter used (Bentin et al., 2007; Lux et al., 2008), even in the case of different uppercase letters made up of different local lowercase letters (Bruyer et al., 2003). Our results partially support this evidence. Thus, while a global advantage was found with both letters, a global interference effect was only observed with the letter “H.” The scarce literature on the effect of letter per se in the global/local processing of hierarchical visual stimuli supports the assumption that the target letter “H” is more quickly identified than the target letter “S” (Lamb and Robertson, 1988; Lamb et al., 1990; Modigliani et al., 2001). This had already been reported initially by Navon (1981), who found a poorer identification of the congruent letter “S” (S_S_) than of the other letters used (H_H_, H_S_, and S_H_). Similarly, the results observed in the present study in the congruent condition show that the target letter “H” (H_H_) was identified more rapidly that the letter “S” (S_S_) both in the global and the local tasks. As we hypothesised, the shorter RTs in identifying the letter “H” versus the letter “S” may be interpreted considering the law of linear periodicity (Uttal, 1975) with regard to the visual perception of form. Intriguingly, in our incongruent condition for the local task, the target letter “_S_” (H_S_) was identified more rapidly than the target letter “_H_” (S_H_), while no differences were found between these letters in the global task. The same result was obtained by Lamb and Robertson (1988). We might interpret, as an explanation for the shorter RTs for the letter “_S_” in the local task (H_S_), the different frequency of use in European Spanish of the letters presented. It is worthy of note that “S” (7.56%), in contrast to “H” (0.66%), is one of the most frequently used letters in European Spanish (Alameda and Cuetos, 1995). Thus, in the local task the target “_S_,” due to its higher familiarity abolishes the expected predominant influence of the global “H”. However, the different frequency of use of letters in the Navon task would not explain those (similar) findings of Lamb and Robertson (1988) with English-speakers, since in English letters “H” and “S” have similar frequency of use (4.74% and 6.63%, respectively, Grigas and Juškevičienė, 2018). An alternative explanation may be derived from the configuration of hierarchical stimuli. As in the study by Lamb and Robertson (1988), the letter H_S_ used in the present study was composed of fewer elements (12) than the letter S_H_ (14), which was necessary to compose the straight and curved strokes in the letters “H” and “S”, respectively. This configuration of the letter “H” with a fewer number of elements might imply that both congruent (H_H_) and incongruent (H_S_) conditions display an equal salience of global and local levels (Yovel et al., 2001). This would lead to similar ease in identifying the global and local letter, which in turn would explain why the target letter “H” (H_H_) in the congruent condition was more rapidly identified in both tasks, and why the local target letter “_S_” was identified more rapidly in the incongruent condition (H_S_). In any case, in the incongruent condition no differences were observed in the global task. In other words, the faster processing of straight strokes seems to be enhanced when both letters of the hierarchical compound were congruent, whereas that the confluence of both straight and curves strokes in the incongruent condition abolished this facilitation. Thus, the faster processing of local “_S_” (Hs) vs. “_H_” (S_H_) could be related to the equal salience of the letter “H” in both global and local levels.

To sum up, though the global advantage is independent of the letter used, the global interference is not. Therefore, the hierarchical letters “H” and “S” in the incongruent condition exerted a modulating influence on the interference effect. In particular, the results presented herein replicate the findings of Lamb and Robertson (1988) and, although further research is needed to provide additional evidence in this same direction and to assess the possible influence of letter frequency on the GPE, we think that the shorter RTs found in both tasks in the congruent and control conditions for the letter “H” may be due to a greater predisposition of the visual system for processing straight lines than for curved lines (Uttal, 1975). In addition, equal saliency (Yovel et al., 2001) of global and local levels in the large letter “H” (H_H_ and H_S_) seems to justify the rapid identification of the local “_S_” in the incongruent condition. In any event, in order to further understand the modulating effect of the letter per se on the GPE, it would be necessary in future experiments to compare curved (e.g., “S”, “U”) and straight line (e.g., “H”, “F”) letters systematically also considering their frequency of use (e.g., “S” and “H” with high frequency and “U” and “F” with lower frequency, according to Grigas and Juškevičienė, 2018, in the English language).

#### Understanding the Modulating Effect of Sex and Women’s Hormonal Status on the GPE

RT analysis in the present study revealed significant differences between the sexes: men responded more rapidly in all tasks and conditions than women. These results match the findings of Lee et al. (2012), who, while noting no differences in accuracy, found shorter RTs in males than in females in a divided attention task. In the current study we observed a generalised GPE, in males as in females. It should be noted that the global interference effect found in the group of females disappears when we examined separately the different hormonal status groups, revealing both symmetric global and local interference effects. Additionally, global processing facilitation was not found to be greater in men than local processing facilitation in women, thus contradicting some results from earlier studies with the Navon task (Roalf et al., 2006; Müller-Oehring et al., 2007; Razumnikova and Volf, 2011) and with the Kimchi-Palmer task (Scheuringer and Pletzer, 2016). In the latter task, participants had to decide, using hierarchical geometric figures, whether the standard figure could be better identified according to its local elements or its global shape (Kimchi and Palmer, 1982). In this regard, findings from the present study are in accordance with those from Basso and Lowery (2004) using the Kimchi-Palmer task, and with those from Pletzer et al. (2014) using the Navon task, who did not find a global bias in males or local bias in females.

Considering hormonal status, unlike some previous studies (Pletzer et al., 2017; Pletzer and Harris, 2018), we found differences among males, luteal women, follicular women and hormonal contraceptive users performing the Navon task. Related to this, in the original study by Pletzer et al. (2014) differences were indeed found among these groups, such as that luteal women showed a narrower global advantage than men, follicular women and hormonal contraceptive users. In the present study, most of the significant between-participants effects were seen during the local task, making this task seem more sensitive to hormonal condition than the global task. In particular, men were found to have local advantage over follicular women reflected in shorter RTs. Moreover, *d’* index analyses showed men to have a higher local advantage over hormonal contraceptive users, and also for luteal women to experience a local advantage over both follicular women and hormonal contraceptive users. The greatest local advantage for luteal women in comparison to the other female groups may reflect in some manner the reduction in global advantage indicated by Pletzer et al. (2014) in this group of women. Likewise, RTs also showed greater interference during the local task in follicular women and hormonal contraceptive users vs. males, and greater interference during the global task in luteal women vs. males. Thus, in contrast to both follicular women and contraceptive users, luteal women could make a more thorough exploration of the irrelevant local level during the global task.

One limitation in the present study is the lack of registered data for the sexual hormone levels of participants. In any event, it is known that males have elevated testosterone levels in comparison with women, while follicular women and luteal women should not differ in their concentration of this hormone (Dabbs and de La Rue, 1991). It is possible, therefore, that the results reflect that, the higher the level of testosterone, the better the performance of the Navon task. Moreover, as indicated in the Introduction, it is also known that luteal women have higher levels of progesterone than women in follicular phase of the menstrual cycle (Reed and Carr, 2018). The results of the present work, showing that luteal women displayed greater local advantage than the other two groups of women, are congruent with those findings of Pletzer et al. (2014) and Pletzer and Harris (2018) which support that higher levels of progesterone can be associated to the greater facilitation of the local processing. This fact, considering too that during the global task luteal women presented greater local interference than men, may support the negative correlation reported by Pletzer et al. (2014) between global advantage and high levels of progesterone present during this phase. Such findings are complementary to the differences found between males and females in relation to other cognitive functions such as visual-spatial capacity or emotional memory (Voyer et al., 1995; Herrera et al., 2019). On this subject, Herrera et al. (2019) consider the locus coeruleus-norepinephrine system an ideal candidate to mediate sex differences in cognition, including those related to visual global and local processing. In fact, a common factor of the differences reported in several cognitive functions between males and females might be, according to these authors, the different processing bias (i.e., holistic for males and analytic for females) that both sexes show while performing complex cognitive tasks.

In sum, the results of the present study do not actually point to global processing facilitation in men and to local processing facilitation in women, but reveal shorter RTs in men as compared to women in the Navon task. Analyses of the discrimination index *d’* have been crucial to reveal differences in women’s hormonal states, showing that luteal women present a *d’* index in the congruent condition during the local task, which is similar to men and greater than that of follicular women and hormonal contraceptive users. We interpret that differences between men and women are due, at least partially, to changes in women according to their hormonal status, since luteal women seem to explore more thoroughly irrelevant local information in the global task. In a broader sense, we suggest that probably luteal women show an accentuated tendency to rely on finer grained information contained in complex figures, displaying preferentially an analytical processing style in visual perception, as previously pointed out by Pletzer et al. (2017). In future research it will be convenient to use biological markers in order to verify the hormonal status in female participants (via commercial ovulation tests) and to assess sex hormone levels by saliva or blood sampling in order to contrast our findings.

### Letter and Sex Are Variables to be Taken Into Account in Studies on Global/Local Processing of Hierarchical Visual Stimuli

In the present work, the influence of letter identity and the differences between men and several groups of women in different hormonal status during global/local processing was studied. Results for RT and *d’* index showed that letter and sex/hormonal status did not interact in relation to global/local processing, but did modulate the GPE independently.

The study of letter as a factor highlighted that stimulus design may be relevant and it should be taken into account in studies on global/local processing of hierarchical visual stimuli. In the present work the letters “H” and “S” were defined, the first as consisting of straight strokes and the second as consisting of curved strokes. Though identical in size, owing to the different letter-form in each case, the “H” was made up of 12 elements and the “S” of 14 elements. In general, different authors have opted in their analyses to average the RTs for each of the different letters used. An alternative that is suggested by the present work is the use of global letters of identical size, of equal stroke type and comprising the same number of local elements. In this regard, the stimuli considered could be those selected by Evert and Kmen (2003), who used the consecutive hierarchical letters “H” and “I” comprising 18 and 17 elements, respectively. These stimuli might be edited to contain a similar number of elements without altering their configuration, they would have very similar characteristics differing only by a 90° rotation, and therefore no differences between both letters would be evident during the global/local task. However, it is appropriate to underscore the use of letters with straight and curved lines, such as the consecutive letters “C” (11 elements) and “D” (14 elements), presented by Bentin et al. (2007) to a patient with congenital prosopagnosia and global processing impairment. We further think that those studies on visual hierarchical stimuli processing which use stimuli of a different nature such as numbers (Slavin et al., 2002), meaningful or meaningless drawings (Poirel et al., 2008), it may be of interest to analyse the potential effect of stimulus identity.

The present study also highlighted the modulating effect of the sex variable in studies on the GPE by means of the Navon task with letters as hierarchical stimuli, as well as the pertinence of conducting hormonal studies allowing us to establish objective correlates with the performance of the Navon task in different groups of women. Therefore, future studies should conduct ovulation tests allowing us to confirm participants’ phase in the hormonal cycle, and hormonal testing such as that incorporated by Pletzer et al. (2014), that allow us to objectively determine the influence of sexual hormones in global/local processing. Given the modulating effect of sex on the GPE found in the present work, studies on the visual processing of hierarchical stimuli should take this variable into account in the experimental design and in conducting the pertinent statistical data analysis.

Lastly, we would stress that the *d’* index revealed significant differences among the groups of women in the local task. The *d’* has rarely been analysed as a dependent variable in studies involving the Navon task, and it has only been used occasionally as a general discrimination index for global/local processing for subsequent comparison with the facial recognition discrimination index (Hills and Lewis, 2009) or in the analysis of event related potentials (Flevaris et al., 2014). We would like to point out that it would be convenient to analyse the *d’* as an indicator of performance in future studies involving the Navon task, especially in studies in which sex differences are analysed taking the menstrual cycle into account.

In conclusion, we think that letter and sex differences according to hormonal status are modulating variables that are relevant to the GPE and should be taken into account in any study on global/local processing, including research on neural correlates in visual processing of hierarchical stimuli (of interest, are event related potential studies such as those carried out by Flevaris et al., 2014 and Iglesias-Fuster et al., 2015 and the fMRI study carried out by Valdés-Sosa et al., 2020). We also maintain the view that the variables letter and sex according to hormonal status should be taken into account in studies analysing global/local processing in relation to face processing (Behrmann et al., 2005; Bentin et al., 2007; Duchaine et al., 2007a,b; Busigny and Rossion, 2011), reading skills and developmental dyslexia (Schmitt et al., 2019) as well as in other studies on the GPE at different stages of the life cycle, specifically during typical ageing (Lux et al., 2008; Agnew et al., 2016) and in neurodegenerative disorders (e.g., Alzheimer’s disease, Slavin et al., 2002; Pike, 2017), in this case for the purpose of characterising cognitive degeneration markers allowing early detection and neurocognitive intervention.

## Data Availability Statement

The datasets presented in this study can be found in online repositories. The names of the repository/repositories and accession number(s) can be found below: https://osf.io/xjmna/.

## Ethics Statement

The studies involving human participants were reviewed and approved by the Ethics Committee of the University Autonomous of Madrid (Code: CEI-71-1271). The patients/participants provided their written informed consent to participate in this study.

## Author Contributions

JI and EO: conceptualization, project administration, funding acquisition, writing – review and editing, and supervision. AÁ-SM, JI, and EO: investigation, data collection, visualization, and writing – original draft. AG and EO: methodology. AG: formal analysis. All authors contributed to manuscript revision, read, and approved the submitted version.

## Conflict of Interest

The authors declare that the research was conducted in the absence of any commercial or financial relationships that could be construed as a potential conflict of interest.
